# A County Hospital

**Published:** 1887-12-17

**Authors:** 


### A County Hospital.

By Our Special Commissioner.

Sketches of three well-known hospitals are conspicuous on the outer page of this Journal. One of them, the "Norfolk and Norwich," is the subject of the present article. There is very much, doubtless, in the experience of every institution of the sort which is common to them all; but, on the other hand, there are many points of divergence, of which, to the non-professional visitor, the most striking is the dissimilarity in the characteristics of the patients at a metropolitan hospital like St. Thomas's, and a county hospital like that at Norwich. The latter is at once a type and a model of its class—a type because nearly every important town in the agricultural districts of England has, fortunately, its own hospital (though some of them are very modest "cottages," indeed); and a model because for years past circumstances have given to this institution a peculiar and an enviable prominence. Its long line of skilful surgeons, and the completeness with which its books of cases have been kept for a considerable period are well known to those interested in hospital work. For the last four years, too, it has enjoyed the advantage of a building which combines those architectual and sanitary improvements of which experience urges the adoption, to a greater degree than many a hospital in larger and more important towns. Norwich is an ancient city, and the hospital is an old institution. It dates from 1772, and the first building is still in use. A hundred years ago, as well as to-day, the citizens exhibited a thoroughness and a zeal in the cause of the suffering poor, which other towns might do well to emulate. In a little over two years the first hospital was projected, built and opened; and when, a century later, it was shown that, with all the additions that had from time to time been made, it was insufficient to meet the demands upon it, there was no lack of friends—from the Earl of Leicester, with a princely donation of £15,000, down to the working men of the city—to help. This is a Royal hospital, too, in a special sense. The Duke of Connaught, when at Norwich with his regiment in 1875, inspected it and became a donor, and eight years later visited the city, with the Duchess, to open the completed building. The Heir-Apparent, with the Princess of Wales, attended a meeting, in 1876, when first the project of the new building was mooted, and three years later laid the foundation-stone. His Royal Highness and the Princess are donors, and the patron and patroness of the hospital respectively; for the Prince takes a special interest in all matters affecting the county, and prides himself on being a Norfolk landowner.

The hospital itself is an imposing building architecturally, and stands in a pleasant and elevated part of the city on the main road to London. Its shape is somewhat that of the letter H, the centre part containing the administrative office, and each side consisting of a two-storied pavilion, the second floors being exactly similar in arrangement to the first. At the back of the administrative block is the operating theatre, a large and well-lighted room. In this part of the hospital also are some small apartments, occupied by the subjects of the operations, or by very critical cases—especially patients suffering from mental diseases—who require in a marked degree quiet and constant attention. For accidents, too, these rooms, some of which contain one, some two beds, are very valuable. There were three or four casualties in these rooms when the writer visited them. One man was just getting over a perfect series of fractures, the result of a machinery accident, and spoke very highly of the comfort, during his most painful days, of the quiet of a bedchamber all to himself. Of this class of cases the hospital has its fair share, to which the works in the city and the use of agricultural implements in this county contribute by far the larger proportion. Three hundred injured people were admitted as in-patients last year, upon eight of whom amputations were necessary. The wards are, of course, large, airy, and bright; nothing less could be expected in so modern a building, and the 220 beds are generally filled. Thoughtful friends in the city and the neighbourhood keep up a good supply of cheerful flowers; and the heating arrangements consist of a couple of therm-hydric stoves in each ward. In the corridors and staircases the temperature is maintained by hot-water coils. A pleasant part of the hospital is the glass-covered way leading from one wing to the other on the second floor, which makes a pleasant lounge for the convalescents in the warm weather. It is somewhat strange that in designing the hospital, a lift for patients does not seem to have been thought of—at any rate, none is provided—but the want has been to a great extent remedied during the past year, by the gift by Mr. E. Copeman of an invalid carrying chair of the latest pattern. There are thirty children's beds, some of which are often empty, not for long, for a large number of medical cases—hip and joint diseases especially—are sent in from the agricultural districts. Under-feeding, parental ignorance, and want of milk as an article of everyday food, are the most prolific causes of the illness of these little ones, and account also for not a few of the diseases of the adult patients. A carefully compiled analytical table, with the diseases arranged according to the nomenclature of the Royal College of Physicians, is a feature of the annual reports, and from these may be seen how many patients come from the city, how many from the country, their age, and the result of their treatment last year. For example, of the 1,124 in-patients, 563 belonged to the town, and 561 to the district outside. The Norwich Hospital has so long had a reputation for the skill of its surgeons in the treatment of stone—a disease which is more prevalent in Norfolk than in any other county—that one is rather surprised to see that last year only nineteen cases were admitted, and but fifteen operations performed. For some unexplained reason, the number of patients suffering from stone has been decreasing. Years ago seventy or eighty admissions per annum were not rare; now the average is very near the figures just quoted for 1886.

A stroll through the wards, in the quiet of a fine autumn afternoon, gives one a very favourable impression of the institution and its management; and the rows of recumbent figures compel a mental comparison between the patients here and those in a London hospital. Somebody may say, "suffering leaves the same traces on the features, whether the sufferer has lived in the New Cut, or on the broad lands of a Norfolk farmer." The remark is only partly true. Pain draws all faces alike—cockney or countryman, prince and peasant—but one sees a great difference between the patients here and in the metropolis; accounted for, it is probable, by the fact that there is little or no over-crowding, and an abundance of fresh air. This is especially noticeable amongst the children, whose little faces are very seldom so preternaturally old-looking as their counter-parts in a London hospital; though on the other hand, they seem duller and altogether less intelligent than town children. The out-patients, too, present collectively a very different sight from that which meets the eye of a visitor to a similar department in a London hospital. Wednesday is the out-patients day at Norwich, and the people come from a very large area—by railway, by farmers' carts, and by the economical method, known as shanks's pony. The out-patients' building is the old hospital, is connected with the more modern structure by a glass-covered way, and stands to the right of the main edifice. Here also is the famous museum, and, on the upper floor, the nurses' home. The latter insttution, too, is in a thriving way, and has done much to improve the standard of nursing in the country, besides also bringing in no considerable sum to the hospital revenues. Last year, in spite of the fact that the services of the members were given gratuitously to the inmates of the Norwich Blind Asylum during a severe outbreak of scarlet fever, the fees for nurses' services amounted to £344 odd. The out-patients number some five thousand every year, and the cases generally occupy the physicians and surgeons in attendance from eleven o'clock to three every Wednesday. The gathering here each week is scarcely so motley as at the hospitals in London and the very large towns, but the difference between the city and country patients is very marked. The absence of a coffee-bar, or any accommodation for supplying refreshments of a cheap kind strikes a visitor, who is accustomed to seeing these conveniences doing an active business in the out-patients' halls in London. One would think that the people, coming as they do from considerable distances, would have welcomed something of the sort. Some time ago the Dispenser, Mr. W. G. Crook, and others, took the matter up, and their conversation with the patients seemed to show that a stall where cheap refreshments might be purchased would be esteemed a boon. Accordingly one was established, and continued in operation for a month or two; but as the largest takings on any one day were one and ninepence, it was very obvious that no contractor could afford to keep up the stall, so it was abandoned. In all other respects, however, the convenience of the out-patients seems to be consulted in every way; nor do they, as a body, have any complaints to make. The very poor people who come from a distance find the cost of the journey somewhat heavy, but when possible they are supplied with medicine for a longer period than a week. Even this, however, does not prevent them from discontinuing their attendance, in many cases, before a cure is effected, with the result that they have to reappear amongst the out-patients, and, in the end, to make more journeys to the hospital than would have been necessary had they only persevered from the first. The patients, in and out alike, have every reason to be grateful to the munificence which has provided such a refuge in sickness; and the citizens of Norwich and the people of Norfolk to be proud of an institution which is a model of its class.

